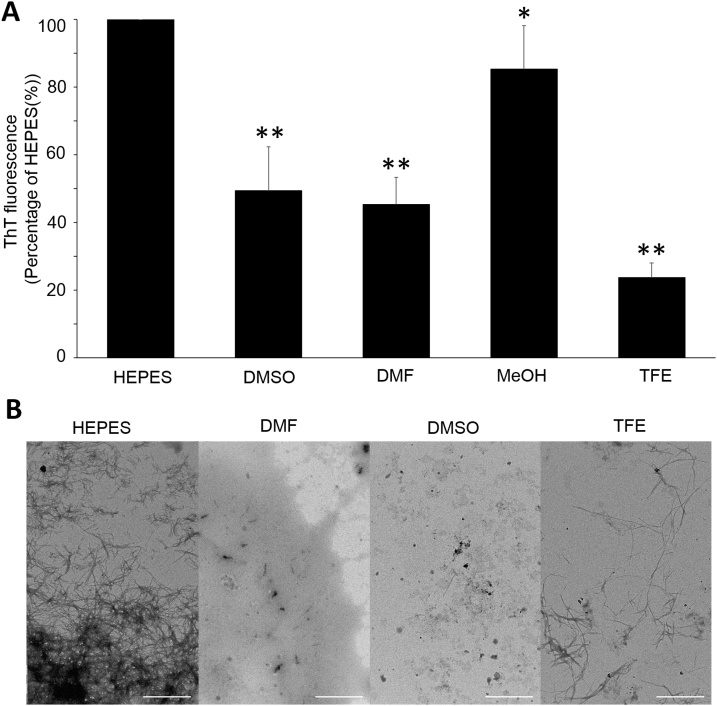# Amyloid-specific extraction using organic solvents

**DOI:** 10.1016/j.mex.2019.100770

**Published:** 2020-01-27

**Authors:** Junichi Kamiie, Naoyuki Aihara, Yu Uchida, Daiki Kobayashi, Yutaka Yoshida, Takeshi Kuroda, Motoharu Sakaue, Yutaka Sugihara, Melinda Rezeli, György Marko-Varga

**Affiliations:** a Laboratory of Veterinary Pathology, School of Veterinary Medicine, Azabu University, Sagamihara, Japan; b Omics Unit, Niigata University Graduate School of Medical and Dental Sciences, Niigata, Japan; c Department of Structural Pathology, Kidney Research Center, Niigata University Graduate School of Medical and Dental Sciences, Niigata, Japan; d Division of Clinical Nephrology and Rheumatology, Niigata University Graduate School of Medical and Dental Sciences, Niigata, Japan; e Laboratory of Veterinary Anatomy II, School of Veterinary Medicine, Azabu University, Sagamihara, Japan; f Division of Clinical Protein Science & Imaging, Department of Biomedical Engineering, Lund University, Lund, Sweden

**Keywords:** Amyloid-specific extraction from FFPE specimens using organic solvents, Amyloid, Organic solvents, Formalin-fixed, Paraffin-embedded, Protein extraction

## Abstract

Typing of amyloidosis by mass spectrometry (MS) based proteomic analysis contribute to the diagnosis of amyloidosis. For MS analysis, laser microdissection (LMD) is used for amyloid specific sampling. This study aimed to establish a method for selectively extracting amyloids from formalin-fixed, paraffin-embedded (FFPE) specimens by organic solvent instead of LMD. The extracts using dimethyl sulfoxide (DMSO), dimethylformamide (DMF), methanol, trifluoroethanol (TFE) or hexafluoro-2-propanol from FFPE brain of alzheimer’s disease mouse model generated protein bands on SDS-PAGE, and Aβ was identified in the extract of DMF using mass spectrometry. The extract using DMSO from the kidney of a AA amyloidosis patient produced a protein band in SDS-PAGE. This protein band was identified to be serum amyloid A (SAA) by Western blotting and mass spectrometry. Circular dichroism spectrometry revealed that the secondary structures of Aβ and transthyretin were converted to α-helices from β-sheets in TFE. Our results suggest that organic solvents can extract amyloids from FFPE specimens by converting their secondary structure. This method could eliminate the LMD step and simplified amyloid typing by MS analysis.

• DMSO, DMF, methanol, TFE and HFIP can extract Aβ specifically from the FFPE brain of a Alzheimer’ disease mouse model.
• DMSO can extract SAA specifically from a FFPE section of AA amyloidosis.
• Secondary structures of Aβ and transthyretin converted from β-sheet to α-helix in TFE.

## Graphical abstract

**Figure:**
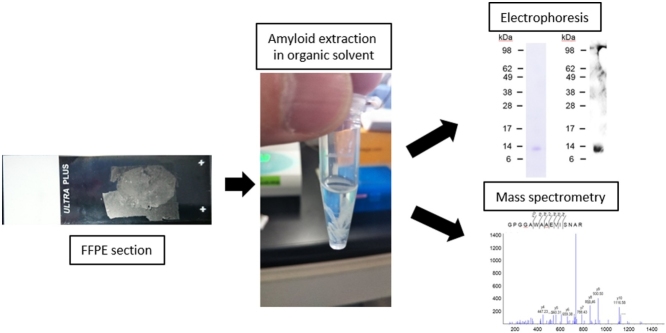

**Specification Table**

**Table:** 

Subject Area:	Medicine and Dentistry
More specific subject area:	Pathology
Method name:	Amyloid-specific extraction from FFPE specimens using organic solvents
Name and reference of original method:	Laser microdisection and mass spectrometry based proteomic analysis
Resource availability:	Laser microdisection and mass spectrometry based proteomic analysis

## Method details

Amyloidosis is caused by the extracellular deposition of amyloid fibrils formed by amyloid precursor proteins [1,2]. In total, 36 and 10 amyloid precursor proteins have been identified in tissue deposits in humans and animals, respectively [1]. However, the mechanism by which amyloid precursor proteins aggregate into amyloid fibrils in diseased tissues is not fully understood [3].

The clinical course and prognosis of amyloidosis is dependent on the type of amyloid protein deposited in tissues [1,2]. Accordingly, the typing of amyloid is an important issue for the management of amyloidosis. Laser microdissection (LMD) and mass spectrometry (MS) based proteomic analysis for amyloid typing has been developed on formalin-fixed, paraffin-embedded (FFPE) tissues are routinely used for human pathological diagnosis [[4], [5], [6]]. In the amyloid typing method with MS analysis, amyloid deposits were retrieved from FFPE tissue using LMD by pathologist [[4], [5], [6]]. LMD requires an expensive device and advanced expertise in pathology. The selectivity for amyloid extraction depends on the accuracy of excising the deposit via LMD. Therefore, a chemical method for selective extraction of amyloid from FFPE without LMD could reduce the laborious LMD step and simplify the amyloid typing using MS analysis.

Protein extraction from FFPE specimens is considered difficult due to the presence of chemical cross-linking [7]. Interestingly, some studies have succeeded in the selective extraction of amyloids from FFPE samples [[8], [9], [10], [11]] and have suggested that amyloid is resistant to fixation with formaldehyde. These studies used several surfactants or denaturants to extract amyloid.

It is well known that the β-sheet structure of amyloid influences amyloid fibril formation [[12], [13], [14]]. We hypothesised that decreasing the β-sheet structure of amyloid can reduce amyloid fibrils and solubilise amyloids from FFPE tissues. The α-helix content of most proteins is increased by incubation in organic solvents [[15], [16], [17]]. It is expected that the β-sheet structure of amyloids decreased by converting it to the α-helix structure in organic solvents. In this study, we aimed to elucidate the secondary structure of amyloid in organic solvents and establish an amyloid extraction method for FFPE samples using organic solvents.

### Extraction of amyloids from FFPE sections of the brain of a Alzheimer’s disease mouse model

To demonstrate the extraction of amyloids from FFPE tissues, we used the 5XFAD mouse brain. 5XFAD mouse is an animal model of Alzheimer’s disease [18]. It overexpressed amyloid precursor protein, and a high deposition of amyloid fibrils formed with Aβ1–42 was observed in the brain. We aimed to demonstrate the extraction of Aβ1–42 from the FFPE brain of 5XFAD mouse.

FFPE brains of a 5XFAD mouse and C57/B6 mouse (control) were provided by Dr James Ken Chambers from the University of Tokyo. Twelve-month-old mice were used, and 10-μm-thick sections were cut from the FFPE brains of 5XFAD and C57/B6 mice. On glass slides, these sections were dewaxed with hexane. After drying at room temperature, the area of each specimen was calculated using photography and ImageJ (https://imagej.nih.gov/ij/). Six sections of a brain (area 16- mm^2)^ on a slide were scratched using a surgical knife and transferred to a 1.5-ml plastic tube. Next, 1 μl of 100 %DMSO, DMF, MeOH, TFE or hexafluoro-2-propanol (HFIP; WAKO) was added to each 1-mm^2^ specimen placed in individual tubes. All the 6 sections on the slide were scratched into a tube and 96 μl of each solvent was added in it. The tubes were incubated at 37 °C for 16 h followed by centrifugation at 15,000 × *g* for 15 min, and the supernatants (amyloid extract) were collected and subjected to sodium dodecyl sulphate-polyacrylamide gel electrophoresis (SDS-PAGE) and MS.

### SDS-PAGE

The amyloid extracts (50 μl) were dried using a Speed Vac at 60 °C for 30 min and dissolved in 10 μl of Laemmli’s sample buffer. After incubation at 37 °C for 15 min, protein components were electrophoretically separated through a 5 %–20 % gradient polyacrylamide gel using a Tris-tricine system (ET-15S e-Pagel, ATTO, Japan). The proteins on the gel were visualised via silver staining (Silver Quest kit, Invitrogen, USA) or Coomassie Brilliant Blue (CBB) staining (Bio-Safe CBB G-250, Bio-Rad, USA).

### MS for amyloid extract

MS was performed using the DMF extract from the brain of 5XFAD and DMSO extract from the kidney of AA amyloidosis patient. Each extract (20 μl) was dried using a speed vacuum and dissolved in 50 μl of a buffer containing 1.2 M urea and 100 mM Tris-HCl, pH 8.0. The extract was then digested in 0.1 μg of TPCK-treated trypsin (Trypsin gold, Promega, USA) overnight at 37 °C. The tryptic peptides were desalted using a C18 spin column (spin trap C18, GL Science, Tokyo, Japan). The eluates from the C18 column were dried and dissolved in 20 μl of 0.3 % formic acid, and 5 μl of each sample were injected into a nano-flow-LC system (Eksgent nanoLC 415 with ekspert cHiPLC, AB Sciex) coupled with a tandem MS system (TripleTOF5600, AB Sciex). Analysis was conducted in duplicate for each sample in the trap and elute mode using a C18 Chip column (75 μm ×120 mm, Nikkyo Technos) as an analytical column. Mobile phases A and B were 0.1 % formic acid and 0.1 % formic acid in acetonitrile, respectively. Peptides were separated in 20-min gradients from 2 % B to 32 % B at 300 nl/min. MS spectra followed by 10 MS/MS spectra were acquired in the data-dependent mode with a cyclic time of 1.3 s.

Product ion output data were searched against the reference database of the *Mus musculus* UniProt KB database for 5XFAD samples and *Homo sapiens* UniProt KB database (UniProt release 5/29/2015) for clinical specimens with a concatenated decoy database using a locally stored copy of the Mascot search engine (version 2.6, Matrix Science, London, UK). A protein was accepted if peptides passed the identity and homology thresholds of the Mascot algorithm. The false discovery rate against the decoy database was <5 %.

### Clinical specimen

A 50-year-old female with rheumatoid arthritis was diagnosed with AA amyloidosis via renal biopsy prompted by proteinuria. The patient died of pancreatitis, after which autopsy was performed. Renal tissue from autopsy specimens was evaluated in this study. Sequential sections of same FFPE renal tissue were analysed by pathological examination and amyloid extraction study. For pathological examination, FFPE section was stained with haematoxylin–eosin (HE) and Congo red. For immunohistochemistry, sections were incubated with a monoclonal antibody against human SAA (Kyowa Medex Co., Ltd., Tokyo, Japan) at room temperature for 1 h. Peroxidase-conjugated anti-mouse IgG (Histofine Simple Stain MAX-PO (M); Nichirei, Tokyo, Japan) was used as the secondary antibody. Immunoreactions were visualised using 3,3′-diaminobenzidine tetrahydrochloride (DAB Tablet; Wako, Tokyo, Japan). Sections (10-μm thick) of specimen was dewaxed and incubated in DMSO, and the extracts were analysed using SDS-PAGE and MS as described previously. The Institutional Review Board of Niigata University Hospital approved our study.

## Method validation results

Sample preparation in amyloid isolation by LMD requires fourth steps [[4], [5], [6]]: (1) Congo red staining of the section, (2) the identification of amyloid deposits, (3) the dissection of amyloid deposits, and (4) the extraction of amyloid from dissected piece by heating in surfactant solution. If organic solvents could extract amyloid selectively from FFPE section, sample preparation requires only extraction step. In this study, we aimed development of selective and simple amyloid extraction method using organic solvent. To demonstrate the selective extraction of amyloid using organic solvents, we extracted Aβ and SAA from FFPE tissues of the 5XFAD mouse brain and from clinical specimens of AA amyloidosis.

### Amyloid extraction from the 5XFAD mouse brain

**Fig. 1:**
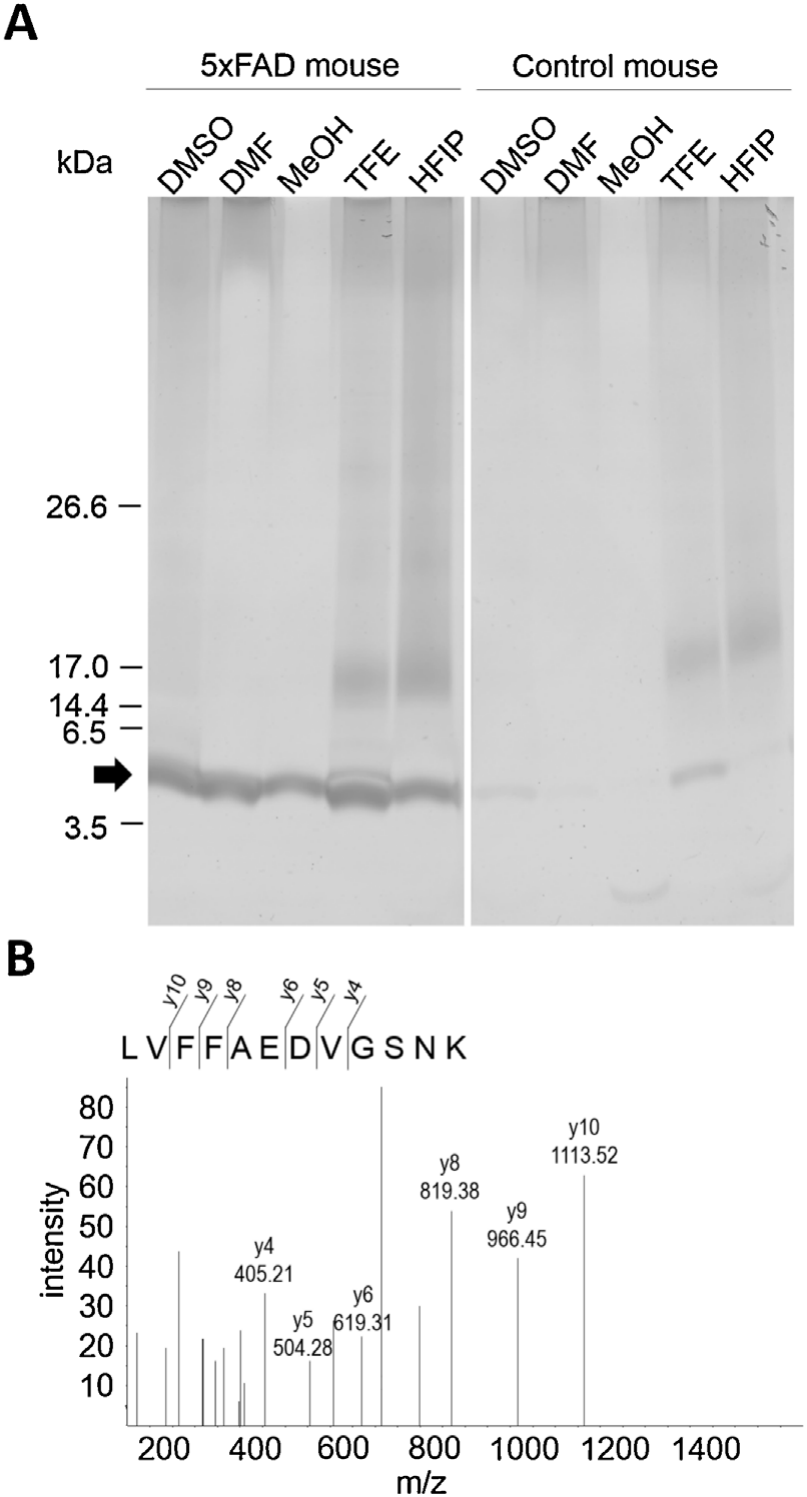
Extraction of amyloid β from 5XFAD mouse brain using organic solvents. (A) SDS-PAGE (silver stain) of extracts from FFPE brains of 5XFAD mouse and C57/B6 mouse with organic solvents. (B) MS/MS spectrum of the doubly charged ion with a specific mass-to-charge ratio 663.34 identified as LVFFAEDVGSNK, which was derived from amyloid βA4.

SDS-PAGE of extracts of 5XFAD mouse brain tissue produced clear bands at approximately 4.5 kDa in all solvents (Fig. 1A). A smear appeared in the high-molecular-weight zone in the DMSO and DMF extracts (Fig. 1A). The TFE and HFIP extracts produced a smear over a broad range and a band at 14.4 kDa (Fig. 1A). The extracts of control mouse brain tissue produced an extremely weak band at 4.5 kDa compared with that observed for the 5XFAD mouse (Fig. 1A). Smears were found in all lanes and a band at 14.4 kDa was detected in the TFE and HFIP lanes for the control mouse brain extract, similar to that observed for the 5XFAD mouse sample (Fig. 1A).

Proteins included in the DMF extract of the 5XFAD mouse brain were identified via MS. Twenty-seven proteins were identified, as listed in Supplemental Table 1. Excluding keratin and trypsin, the protein with the highest score in the Mascot search was Aβ A4, i.e. the precursor protein of Aβ. A peptide sequence of LVFFAEDVGSNK derived from Aβ was detected with a clear MS/MS spectrum of Y-series ions (Fig. 1B).

### Amyloid extraction from a clinical specimen

**Fig. 2:**
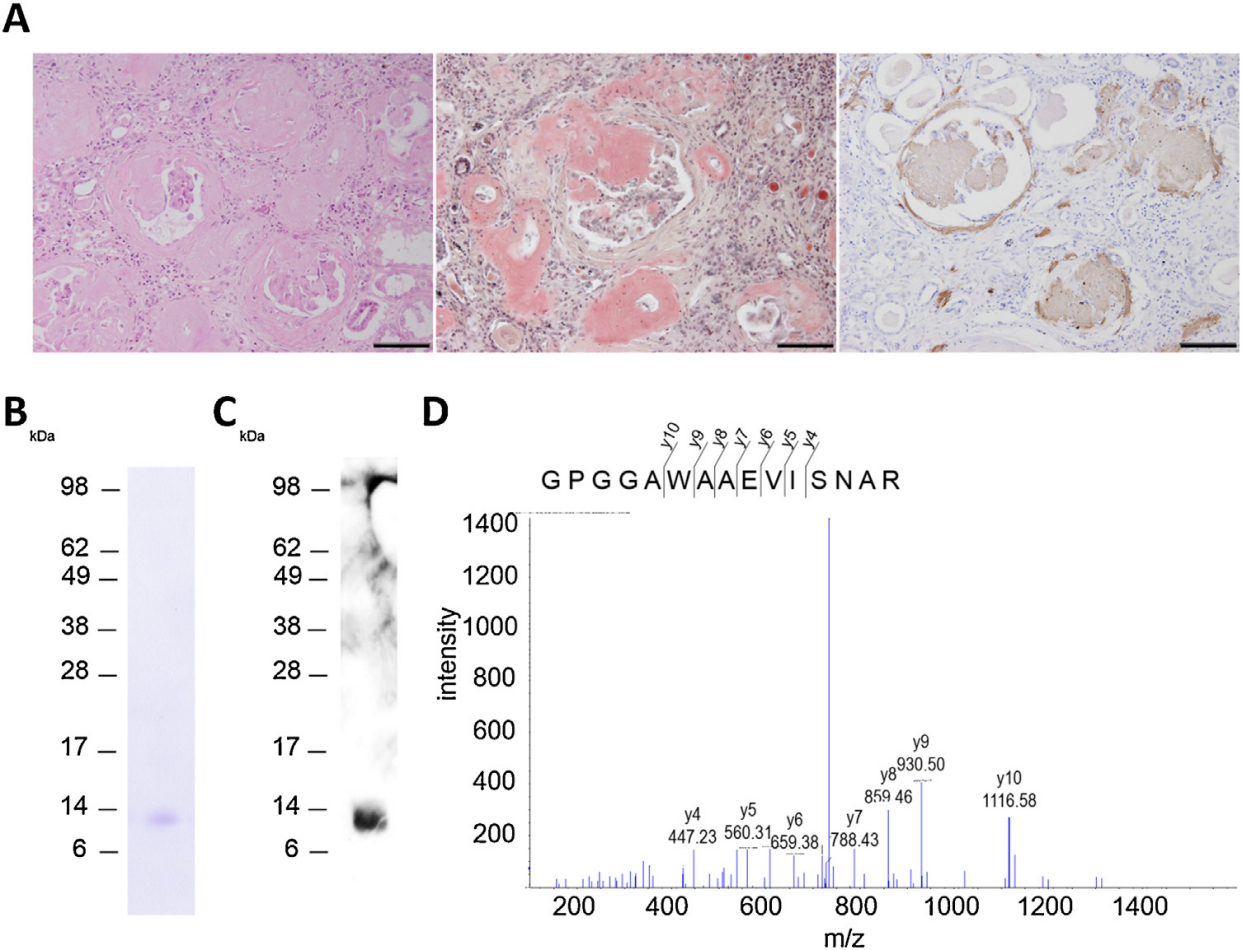
(A) Histopathological findings of the kidney of a patient with AA amyloidosis. HE staining (right) shows amorphous material deposition in glomerular and interstitial tissues. Amorphous materials were stained by Congo red (centre) and are immunohistochemically positive against SAA (left) (scale bar: 100 μm). (B) SDS-PAGE (CBB staining) of the extract from FFPE kidney of a patient with AA amyloidosis with DMSO. (C) Western blotting of the extract against SAA. (D) MS/MS spectrum of the doubly charged ion with a specific mass-to-charge ratio 728.37 identified as GPGGAWAAEVISNAR, which was derived from SAA2.

HE staining revealed severe deposition of eosinophilic amorphous materials in glomeruli, small vessels and interstitial tissue of the kidney tissue from a patient (Fig. 2A). The eosinophilic amorphous material was stained with Congo red dye (Fig. 2A), and it was positive for AA amyloid on immunohistochemistry (Fig. 2A).

The DMSO extract from the kidney of a patient produced a single protein band of approximately 14 kDa in size in SDS-PAGE (Fig. 2B). This protein band was detected using Western blotting against SAA (Fig. 2C). Proteomics analysis identified 38 proteins in the DMSO extract (Supplemental Table 2). The protein with the top score in the Mascot search was SAA2, which was identified by detecting the peptide sequence of GPGGAWAAEVISNAR (Fig. 2D). In addition, serum SAA1 was identified with a high score of 187 in the Mascot search. The protein sequence GPGGVWAAEVISDAR and its missed cleavage sequence (RGPGGVWAAEVISDAR) were detected as the peptides derived from SAA1. The 38 proteins identified via MS analysis are listed in Supplemental Table 2.

### Conclusion

To examine the extraction of amyloids from FFPE tissues, extraction with organic solvents was performed on the FFPE brain of 5XFAD mouse, which overexpress amyloid precursor protein and show high Aβ deposition in the brain [18]. SDS-PAGE and MS indicated that organic solvents could solubilise Aβ deposited in FFPE brain tissues from 5XFAD mice. All organic solvents used in this study extracted an Aβ that produced a band at 4.5 kDa with stronger intensity for 5XFAD mice than for control mice. The TFE and HFIP extracts contained a 14.4-kDa protein, which was considered a nonamyloid because it appeared at the same intensity in extracts from both 5XFAD and control mice. On the contrary, the DMSO, DMF and methanol extracts contained a single protein band at 4.5 kDa and weak smears in the high-molecular-weight area. The clear single protein band at 4.5 kDa on SDS-PAGE coincided with the molecular weight of the Aβ peptide, indicating that DMSO, DMF and methanol can extract the Aβ selectively from FFPE brain samples. Our findings suggested that organic solvents are useful for amyloid-selective extraction from FFPE specimens.

In this study, proteins other than Aβ were identified in the extract from 5XFAD mouse brain via MS. These non-amyloid might be cross-linked by formalin fixation, and they should appear as smears in the high-molecular-weight area on SDS-PAGE.

Organic solvents such as TFE are known as inducers of an ordered structure usually of the helix structure. A considerable amount of research has been performed on the conformation of homopeptides in these solvents [16,17], but information regarding amyloids in these solvents is limited. The secondary structure of the amyloids Aβ1–42 and transthyretin in organic solvents was examined in this study. CD spectrometry illustrated that the secondary structure of these amyloids were converted from β-sheets to α-helix in TFE (supplementary Fig. 1). The ThT assay and electron microscopy showed that the formation of amyloid fibrils by Aβ1–42 decreased in organic solvents, suggesting that amyloid fibrils had dissolved (supplementary Fig. 2). These data suggest that alteration of the secondary structure of amyloids induces fibril reduction in organic solvents.

The proteins in tissue are fixed with formaldehyde via chemical cross-linking, making them difficult to dissolve after fixation. It has been reported that the resistance of the protein that forms amyloid fibrils by chemical cross-linking due to formaldehyde fixation could be related to the amyloid fibril structure, which consists of packed aggregates of β-sheets [8,9]. Therefore, it is conceivable that organic solvents can extract amyloids selectively from FFPE tissues by converting their secondary structure to α-helix. The defining structural element of amyloid fibrils is the cross-β structure [[12], [13], [14]]. Theoretically, organic solvents could extract various amyloids from FFPE specimens by changing the protein structure. We demonstrated the extraction of SAA using DMSO from FFPE kidney obtained from a patient with AA amyloidosis, which was diagnosed by pathological examination and immunohistochemistry. The DMSO extract from a single section of 1- × 1-cm^2^ FFPE kidney showed a protein band clearly in CBB-stained SDS-PAGE gel. The amount of extracted SAA was estimated to be >10 ng based on the fact that detection limit of CBB stain is approximately 10 ng protein. This result indicates the high efficiency and selectivity of this extraction method. Therefore, this extraction method should be useful for small specimens of biopsy sample using highly sensitive methods such as MS spectrometry.

Several studies demonstrated the utility of LMD and MS-based proteomic analysis for typing amyloidosis with high sensitivity and specificity in FFPE clinical biopsy specimens [[4], [5], [6]]. In these studies, amyloid deposits were captured via LMD, whereas amyloids were extracted as peptides following trypsin digestion for MS analysis. The selectivity for amyloid extraction depends on the accuracy of excising the deposit via LMD. The organic solvent extraction method developed in this study reveals the possibility of amyloid-selective extraction from whole sections of FFPE specimens. Because of its high selectivity, it is conceivable that the combination of organic solvent extraction and LMD-MS analysis could identify amyloids deposited in FFPE samples with high precision. The amyloid extraction method using organic solvent instead of LMD could reduce sample preparation step and simplify the MS based amyloid typing.

In summary, we demonstrated the selective amyloid extraction from FFPE specimens using organic solvents, and the extract can be applied to protein analysis including electrophoresis and MS analysis. This novel method should contribute to the study of amyloidosis.

## Declaration of Competing Interest

The authors declare that they have no known competing financial interests or personal relationships that could have appeared to influence the work reported in this paper.

## Figures and Tables

**Figure:**
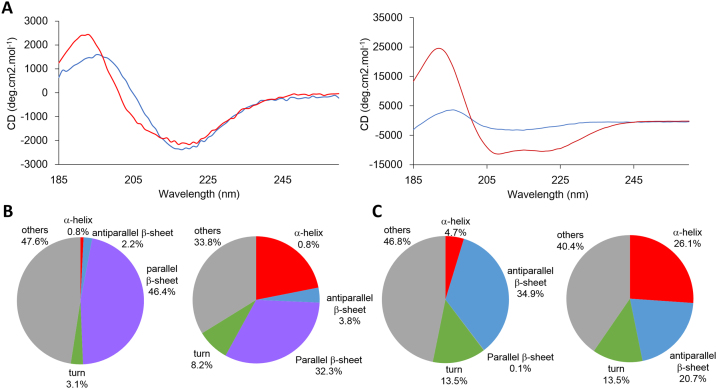


**Figure:**