# Supplementary material for: Amyloid-specific extraction using organic solvents
Source: MethodsX. 2020 Jan 27;7:100770. doi: 10.1016/j.mex.2019.100770 (PMC7046812; doi:10.1016/j.mex.2019.100770)
Supplement: Supplementary file 3 [file mmc3.docx]

Supplementary materials and methods.

*Secondary structure analysis of amyloids in organic solvents via* Circular dichroism *(CD) spectrometry*

Amyloid β (Aβ) 1–42 (Peptide Laboratory, Osaka, Japan) and transthyretin (Wako, Tokyo, Japan) were each dissolved to a concentration of 0.2 mg/ml in 20 mM Tris–HCl, pH 8.0 or 100% trifluoroethanol (TFE; Wako). We used TFE as the solvent for CD spectrometry analysis because it displays extremely low absorbance over the wavelength range used in this study. Spectra were measured on Chirascan (Applied Photophysics) over the far UV wavelength range of 185–260 nm at 15°C in a 10-mm path-length cell. Results are expressed as molar ellipticity (deg.cm^2^.mol^−1^). Secondary structures were determined from the CD spectra using the BeStSel algorithm (<http://bestsel.elte.hu/index.php>; Micsonai et al. PNAS 112:E3095-103 (2015)).

*Thioflavin T (ThT) assay*

Aβ1–42 was dissolved in 100 mM HEPES–NaOH, pH 7.4 at a concentration of 0.2 mg/ml. Fibrillation was induced via sonication followed by incubation at 37°C for 5 days without agitation. Formed amyloid fibrils were collected via centrifugation at 21,000 × *g* for 30 min. Supernatant was removed, and the pellet was dissolved in HEPES buffer, 100% dimethyl sulfoxide (DMSO; Wako), 100% dimethylformamide (DMF; Wako), 100% methanol (MeOH; Wako) or 100% TFE and incubated at 37°C for 20 h. ThT was added to 200 µl of samples at a final concentration of 50 mM. ThT fluorescence was measured at 480 nm (λ*_ex_* = 450 nm) at 25°C using POWERSCAN MX (DS Pharma Biomedical, Osaka, Japan).

*Electron microscopy*

For electron microscopy, 10 µl of samples of dissolved Aβ1–42 fibrils in each solvent were diluted 10-fold with distilled water and placed on copper grids covered with a carbon film (Nissin EM, Tokyo, Japan). The grids were stained with 2% EM Stainer (Nissin EM) and examined using a transmission electron microscope (JEM-2100).

*Statistical analysis*

Statistical significance between two groups was analyzed by t-test (two-sided). Differences were regarded significant with P<0.05. Data are shown as mean ± SEM.

**Supplementary results**

*Structural characterisation of amyloids in organic solvents*

Structural characterisation of Aβ1–42 and transthyretin was performed via CD spectroscopy. Supplementary figure 1 shows the far UV CD spectra of both proteins. The spectra of both proteins differed according to incubation in Tris–HCl buffer and TFE. The CD spectra of transthyretin in TFE were characterised by the typical α-helix-rich shape with two minima in ellipticity around 208 and 221 nm. The calculation using the BeStSel algorithm suggested that Aβ1–42 was made up of 0.8% α-helices in Tris buffer and 48.6% β-sheets. When Aβ1–42 was dissolved in TFE, α-helices accounted for 21.9% and β-sheets decreased to 36.1% of the peptide structure. The difference in the CD spectra between each environment was more prominent for transthyretin. The structure of this protein comprised 26.1% of α-helices and 20.7% of β-sheets in TFE compared with 4.7% of α-helices with 35% β-sheets in Tris–HCl buffer.

*Characterisation of amyloid fibrils in organic solvents*

Amyloid fibrils formed with Aβ1–42 were dissolved in organic solvents, and fibrils in each solvent were quantified using the ThT assay. In comparison with the findings in HEPES buffer (set as 100%), the fluorescence intensities were decreased to 49% in DMSO, 45% in DMF, 85% in MeOH and 24% in TFE (supplementary figure 2A). The ultrastructure of fibrils in each solvent was observed via electron microscopy with negative staining. The Aβ1–42 peptide exhibited typical amyloid fibrils, which were long and rigid, in HEPES buffer (supplementary figure 2B). In the organic solvents, the density of fibrils was decreased compared with that in HEPES buffer. Short fibrils under 500 nm in length were observed in DMF and DMSO (supplementary figure 2B). However, fibrils in TFE were longer than those in DMF and DMSO (supplementary figure 2B).

**Supplementary figure legends**

Supplementary Fig. 1. The secondary structure of Aβ1–42 and transthyretin in TFE. (A) CD spectrum of Aβ1–42 (right) and transthyretin (left). The secondary structure composition of Aβ1-42 (B) and transthyretin (C) determined using the BeStSel algorithm. In both amyloids, the proportion of α-helix increased and that of β-sheet decreased in TFE.

Supplementary Fig. 2. Quantitation and ultrastructure analysis of amyloid fibrils formed with Aβ1–42 in organic solvents. (A) Thioflavin T fluorescence levels are expressed as the percentage of HEPES fluorescence level (n = 5). Significant differences compared with HEPES are marked with asterisks (*p<0.05, **p<0.05). (B) Ultrastructure of amyloid fibrils in each organic solvent (scale bar: 2 µm).
